# Plasma p-tau217 as a biomarker for Alzheimer's disease: diagnostic and prognostic value in clinical and population-based studies

**DOI:** 10.1055/s-0046-1829032

**Published:** 2026-10-09

**Authors:** Gunter Gerson, Guilherme Nobre Nogueira, Maria Jânia Teixeira

**Affiliations:** 1 Universidade Federal do Ceará, Departamento de Patologia e Medicina Legal, Fortaleza CE, Brazil.; 2 Universidade Federal do Ceará, Departamento de Medicina Clínica, Fortaleza CE, Brazil.

**Keywords:** Alzheimer Disease, Biomarkers, Tau Proteins, Dementia, Biomarkers

## Abstract

**Background:**

Alzheimer's disease (AD) is the leading cause of dementia worldwide, and blood-based biomarkers are emerging as accessible diagnostic tools. Plasma phosphorylated tau at position 217 (p-tau217) has shown promise in early detection.

**Objective:**

To evaluate the diagnostic accuracy, prognostic value, and applicability of plasma p-tau217 in AD compared with other biomarkers.

**Methods:**

We conducted an integrative review on the PubMed, Scopus, Embase, and Cochrane Library databases (2018–2024). Eligible observational and diagnostic studies assessing p-tau217 in plasma were included. Study selection and quality assessment followed the Standards for Reporting of Diagnostic Accuracy (STARD) and Strengthening the Reporting of Observational Studies in Epidemiology (STROBE) guidelines.

**Results:**

We analized 9 studies involving more than 6 thousand participants analyzed. Plasma p-tau217 demonstrated high accuracy (area under the curve [AUC]: 0.86–0.94) for detecting tau and amyloid pathology, outperforming p-tau181. It was effective in diverse populations, asymptomatic individuals, and primary care settings. Longitudinal data suggest predictive value for cognitive decline.

**Conclusion:**

Plasma p-tau217 is a reliable and non-invasive biomarker for AD, with applications in diagnosis, prognosis, and treatment monitoring. Standardization and multicenter validation are necessary before clinical implementation.

## INTRODUCTION

Alzheimer's disease (AD) is the leading cause of dementia worldwide, with a growing impact due to population aging. Its pathophysiology is complex, involving the extracellular deposition of beta-amyloid (Aβ) plaques and the hyperphosphorylation of the tau protein, which culminates in the formation of intracellular neurofibrillary tangles. This pathological cascade precedes the onset of clinical symptoms by years, or even decades, making early diagnosis and the identification of at-risk individuals, central elements in intervention, monitoring, and research strategies. 1 2

The identification of sensitive and specific plasma biomarkers has become a priority, especially given the limited access to tests such as positron-emission tomography (PET) or cerebrospinal fluid (CSF) analysis, which are considered gold-standard methods. 3 Among the emerging candidates, tau protein phosphorylated at position 217 (p-tau217) stands out as one of the most promising. 4 Plasma p-tau217 shows a high correlation with the status of tauopathies in the brain, and it is detectable even before tau-PET positivity and before the appearance of significant clinical symptoms. 5 6

Comparatively, p-tau217 has proven to be more sensitive and specific than other subtypes, such as p-tau181, including to distinguish AD from other dementias. 7 8 Its applicability extends to prognosis and the screening of asymptomatic individuals at increased risk for cognitive decline. 9 10 A recent meta-analysis 11 that synthesized data from more than 5 thousand patients has reinforced the high accuracy of p-tau217, with consolidated sensitivity and specificity around 82 to 86% to detect AD pathology. While this meta-analysis quantitatively synthesized diagnostic accuracy metrics of p-tau217, the present integrative review expands upon these findings by incorporating a qualitative analysis across heterogeneous study designs, including primary care cohorts, longitudinal population-based studies, and genetically-defined populations. Additionally, the current review emphasizes real-world applicability, prognostic value, and contextual factors such as healthcare setting and population diversity, which are not fully addressed in prior meta-analytic approaches.

Given this scenario of robust evidence, an integrated analysis is essential. Therefore, the objectives of the predent review are to integrate data from clinical and population-based studies to estimate the predictive value of p-tau217 for dementia, to review its diagnostic and prognostic applicability, and to evaluate its performance in blood compared with p-tau181, tau-PET, and CSF biomarkers.

## METHODS

The current integrative review was guided by the following research question, structured according to the Population, Intervention, Comparison, Outcome, and Timeframe (PICOT) framework: In adults with suspected or at risk of dementia (P), what is the diagnostic accuracy and predictive value of the plasma p-tau217 biomarker (I) compared with tau-PET, CSF, or p-tau181 (C), in the early detection of AD and in the prediction of cognitive decline (O), considering recent clinical and population-based studies (T)?

A literature search was conducted on the PubMed, Scopus, Embase, and Cochrane Library databases for articles published between January 2018 and January 2024. A combination of descriptors and Boolean operators was used to create the search string: ( *p-tau217* OR *phosphorylated tau 217* OR *tau-217* OR *phospho-tau217* OR *tau p217* ) AND ( *Alzheimer disease* OR *dementia* OR *AD* OR *neurocognitive disorder* OR *cognitive impairment* OR *mild cognitive impairment* OR *MCI* ) AND ( *plasma* OR *blood* OR *serum* OR *biofluid* OR *extracellular vesicles* OR *EVs* ) AND ( *biomarkers* OR *diagnostic accuracy* OR *predictive value* OR *prognostic value* OR *early detection* OR *screening* OR *detection* OR *diagnosis* OR *prognosis* OR *monitoring* OR *risk assessment* OR *blood tests* OR *liquid biopsy* OR *circulating biomarkers* ).

Overall, the included studies demonstrated moderate to high methodological quality. Most diagnostic studies adhered to the Standards for Reporting of Diagnostic Accuracy (STARD) guidelines, clearly describing index tests, reference standards, and participant selection. The cohort studies followed the Strengthening the Reporting of Observational Studies in Epidemiology (STROBE) guidelines, with appropriate reporting of study design, follow-up, and outcome assessment.

However, some limitations were identified, including heterogeneity in assay methods, variability in cutoff definitions, and limited reporting of blinding procedures. The longitudinal studies showed greater variability due to potential attrition bias and differences in follow-up duration. Despite these limitations, the overall methodological rigor supports the reliability of the findings.

Original observational and diagnostic studies that evaluated p-tau217 in human plasma or serum with correlation to clinical outcomes, comparative biomarkers, or neuropsychological tests were included. Preclinical studies, reviews, editorials, and articles with incomplete methodological data were excluded. The selection process began with the importation of 768 articles into the Rayyan review tool (Rayyan Systems Inc.). After removing 333 duplicates, the titles and abstracts of 435 unique articles were screened, with 9 studies ^8,1,12–17^ ^,19^ being included after a full-text reading, as illustrated in Figure 1 . This step was performed by two independent reviewers and, in cases of disagreement, a third reviewer was consulted to reach a consensus. Data extraction from the eligible studies was performed using a standardized instrument, and the methodological quality was critically assessed based on the STARD and STROBE guidelines, without applying a formal score but considering the methodological rigor and validity of the methods used. Finally, the data were organized into thematic categories to enable a narrative synthesis that highlights convergences, divergences, and gaps in the literature, ensuring the precision and relevance of the results.

**Figure 1:**
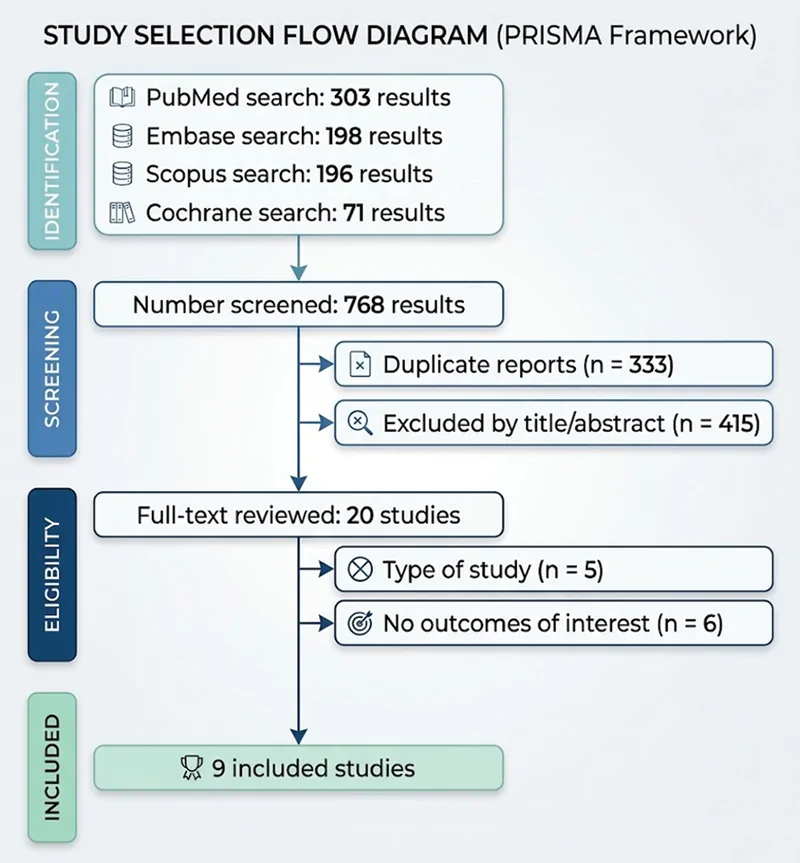
Preferred Reporting Items for Systematic Reviews and Meta-Analyses (PRISMA) flow diagram of study screening and selection.

## RESULTS

The analysis of the selected studies demonstrated the robustness of p-tau217 as a biomarker for AD in different contexts. The findings were grouped into clinical studies focused on diagnostic validation and population-based studies with an emphasis on prognostic value. Table 1 summarizes the characteristics of the studies included.

**Table 1:** Characteristics of the studies included in the integrative review

Author (year)	Study type/Location	Population/N (age in years)	Plasma assay/Biomarker	Comparators and main outcomes	Accuracy (AUC)	Main limitations
Janelidze et al. (2021) 12	Prospective clinical cohort/Sweden	Early cognitive complaints; **N = 1,402** (∼ 71)	p-tau217 (Lilly)	PET-tau, CSF; early detection of tau pathology	**0.91** (versus PET-tau)	Specialist clinic setting; limited generalizability to primary care
Therriault et al. (2023) 13	Clinical cohort/Sweden	Suspected AD; **N = 1,073** (70–72)	p-tau217 (Lilly/Quanterix)	CSF (p-tau, Aβ42/40); diagnostic equivalence	**0.88** (p-tau217), **0.90** (CSF)	High AD prevalence in the cohort may overstate the predictive value
Benussi et al. (2025) 14	Comparative study/Italy	AD versus FTD; **N = 200** (∼ 68)	p-tau217/NfL ratio (Olink/Simoa)	NfL, PET, CSF; etiological differentiation	**0.90** (ratio)	Small sample size for specific FTD subtypes
Zhong et al. (2025) 15	Diagnostic validation/China	Outpatients; **N = 487** (∼ 68)	p-tau217 and Aβ42/40 (Simoa)	CSF, PET-Aβ; accuracy of p-tau217/Aβ42 ratio	**0.91–0.93**	Validation needed in non-Asian diverse populations
Aguillon et al. (2023) 16	Genetic AD study/Colombia	*PSEN1* mutation carriers; **N = 267** (35–50)	p-tau217 (Lilly)	PET-amyloid/tau; correlation with decline	**0.89** (Aβ), **0.92** (tau)	Results specific to E280A mutation; may differ in sporadic AD
Janelidze et al. (2023) 19	Longitudinal study/Sweden	Asymptomatic; **N =7 84** (∼ 71)	p-tau217	PET-amyloid/tau; prediction of PET positivity	**0.86–0.91**	Selection bias (highly-educated/healthy volunteers)
Xiao et al. (2024) 10	Population cohort/China	Community-dwelling; **N = 1,857** (60 + )	p-tau181/217, NfL, GFAP	Cognition; prediction of dementia (5–13 years)	**0.79–0.96** (ML models)	ML-model complexity hinders immediate routine clinical use
Sanford et al. (2024) 8	Multiethnic cohort/United States	Diverse (White/Black/Latino); **N = 580** (∼ 65)	p-tau, Aβ42/40, NfL (Quanterix)	Diagnostic performance across ethnicities	**0.92–0.96** (amyloid)	Potential impact of varying vascular comorbidity rates between groups
Ashton et al. (2024) 17	Primary-care cohort /United Kingdom and Sweden	Suspected dementia; **N = 1,114** (∼ 70)	p-tau217 (ALZpath)	CSF, cognition; accuracy in non-specialized settings	**0.94** (versus CSF)	Implementation logistics and cost-effectiveness in primary care

Abbreviations: Aβ, beta-amyloid; AD, Alzheimer's disease; AUC, area under the curve; CSF, cerebrospinal fluid; FTD, frontotemporal dementia; GFAP, glial fibrillary acidic protein; ML, machine learning; NfL, neurofilament light; p-tau217, phosphorylated tau at position 217; PET, positron-emission tomography; *PSEN1* , *presenilin 1* gene.

Based on the in-depth analysis of the data provided and the interpretation of the generated graphs, it is possible to develop a rigorous and detailed academic description of accuracy (area under the curve, AUC) in the detection of pathologies. This analysis considers the multifaceted influence of the study design employed and the intrinsic characteristics of the investigated populations, using the numerical AUC values as the foundation for the conclusions.

In a large prospective clinical cohort study involving 1,402 patients with early cognitive complaints, Janelidze et al. 12 investigated the role of plasma p-tau217 in the early detection of AD pathology. The study compared plasma levels of p-tau217 with tau positron-emission tomography (PET-tau) and cerebrospinal fluid (CSF) biomarkers, which are considered gold standards to detect tau-related neurodegeneration. Plasma p-tau217 demonstrated a high diagnostic accuracy, achieving an AUC of 0.91 against PET-tau, like cited in Figure 2 . These findings highlight its capacity to detect AD pathology even at the prodromal stages, in which symptoms may be subtle or non-specific. This study 12 provides compelling evidence that plasma p-tau217 could serve as a reliable first-line tool to screen individuals with mild cognitive impairment or subjective cognitive decline, potentially reducing the need for invasive lumbar punctures or costly imaging procedures.

Therriault et al. ^13^ extended this line of research by recruiting 1,073 patients with suspected AD, aiming to directly compare the diagnostic equivalence of plasma and CSF biomarkers. Their results confirmed that plasma p-tau217 performed nearly as well as CSF p-tau and the Aβ42/40 ratio, with an AUC of 0.88 for plasma and of 0.90 for CSF. The narrow difference between these two approaches underscores the growing role of blood-based biomarkers as substitutes for invasive CSF testing. Importantly, the authors 13 emphasized that blood tests may drastically expand diagnostic accessibility, enabling earlier and broader identification of patients who could benefit from specialized assessment and treatment. This equivalence also paves the way to use plasma markers in large-scale clinical trials in which repeated lumbar punctures are impractical.

One of the major clinical challenges in dementia care is distinguishing AD from frontotemporal dementia (FTD), as their early symptoms may overlap significantly. Benussi et al. 14 addressed this by studying 200 patients with AD and FTD, focusing on the diagnostic potential of the p-tau217/neurofilament light (NfL) ratio. Their findings showed that this ratio achieved an AUC of 0.90, substantially improving the discrimination between AD and FTD compared with single biomarkers alone. This study 14 demonstrates that p-tau217 is not only useful to detect AD but also to differentiate it from other dementias with distinct therapeutic and prognostic implications. The integration of p-tau217 with NfL provides a biomarker panel capable of refining etiological diagnosis, thereby improving patient stratification and management.

Zhong et al. 15 performed a diagnostic-validation study in 487 outpatients, a setting that better reflects the real-world clinical practice outside specialized memory clinics. The investigators assessed the diagnostic accuracy of plasma p-tau217 and its ratio with Aβ42, using CSF and PET-amyloid as comparators. Their results revealed high diagnostic performance, with AUCs ranging from 0.91 to 0.93, indicating that plasma measures could reliably replicate the accuracy of gold-standard methods. This study 15 is particularly important because it demonstrated that plasma p-tau217 maintains robust performance in diverse outpatient populations, reinforcing its translational value for broad clinical implementation.

Focusing on genetic forms of AD, Aguillon et al. 16 evaluated 267 carriers of the *presenilin 1* ( *PSEN1* ) mutation associated with autosomal dominant AD (ADAD). This population provides a unique opportunity to test biomarkers in the preclinical phase of AD, since mutation carriers are almost certain to develop the disease. Plasma p-tau217 was strongly correlated with PET-amyloid and PET-tau imaging, and, importantly, it predicted cognitive decline years before the onset of symptoms. These findings highlight the prognostic power of the biomarker to identify pathology and to anticipate disease trajectory. The results also suggest that plasma p-tau217 could become a cornerstone tool in preventive trials targeting high-risk populations.

In another contribution, Janelidze et al. ^6^ conducted a longitudinal study including 784 asymptomatic individuals. The researchers investigated whether plasma p-tau217 could predict amyloid and tau PET positivity before the clinical onset of dementia. The results showed that plasma p-tau217 consistently anticipated PET conversion, with AUC values between 0.86 and 0.91. These findings indicate that p-tau217 is not only a diagnostic marker but also a powerful predictor of preclinical AD. This expands its usefulness to population-level screening and preventive interventions, in which where early detection of at-risk individuals is crucial for the success of emerging disease-modifying therapies.

Xiao et al. 10 took a public health perspective by following 1,857 community-dwelling older adults over a period of 5 to 13 years. Plasma p-tau217 levels were analyzed alongside cognitive tests and clinical data, and advanced machine-learning models were applied to enhance predictive accuracy. Their results demonstrated that plasma p-tau217 could predict the incidence of dementia with AUC values ranging from 0.79 to 0.96. This study 10 is significant because it shows how p-tau217 can be integrated into predictive models to stratify dementia risk in large populations, paving the way for preventive strategies and resource planning in healthcare systems.

Sanford et al. 8 addressed the critical issue of biomarker performance across diverse populations. In a cohort of 580 participants of White, Black, and Latino backgrounds, plasma p-tau217 maintained high diagnostic accuracy (AUC: 0.92–0.96), outperforming p-tau181 and other blood biomarkers. This study underscores the equity of p-tau217 as a diagnostic tool, supporting its use in ethnically-diverse populations in which disparities in dementia diagnosis often exist. By confirming its robustness across groups, Sanford et al. 8 provided evidence that plasma biomarkers can help close diagnostic gaps and contribute to fairer access to early dementia care.

Ashton et al. 17 focused on primary care, an essential setting for early dementia detection. They studied 1,114 patients assessed in the general practice and found that plasma p-tau217 achieved an AUC of 0.94 when compared with CSF biomarkers. This result is striking because it demonstrates that a simple blood test can achieve gold-standard accuracy even outside specialized centers. The authors ^17^ highlighted that implementing plasma p-tau217 testing in primary care could revolutionize the diagnostic pathway by enabling general practitioners to identify high-risk patients earlier and refer them for specialist evaluation, ultimately decentralizing dementia diagnosis and improving access to care.

A notable variation in the accuracy of pathology detection methods is observed depending on the research methodology adopted. Studies categorized as *primary care cohort* and *multiethnic cohort* stand out for presenting the highest mean accuracy values, with AUCs of 0.94 and 0.92 respectively. These results suggest that integrated approaches in primary care settings—often dealing with a wide range of clinical presentations—and studies encompassing the ethnic and cultural diversity of populations may optimize detection capabilities. In contrast, studies with longitudinal designs such as *longitudinal study* and *longitudinal population cohort* showed significantly lower 8 17 mean AUC values, of 0.86 and 0.79 respectively. This discrepancy may be attributed to inherent challenges in tracking individuals over extended periods, including potential heterogeneity in disease progression and difficulties in maintaining methodological consistency.

Additionally, a detailed analysis reveals the critical impact of the characteristics of the studied population on achieving accurate results. Specific populations, such as “Patients with suspected dementia in primary care/N = 1,114” ^17^ and “Diverse population (White, Black, Latino)/N = 580,” ^8^ were associated with the highest mean AUC values, reaching 0.94 and 0.92. These findings reinforce the premise that the careful selection of relevant and well-defined population groups is a determining factor for diagnostic success, enabling the application of detection methods that are better calibrated and more sensitive to the specificities of these groups. Conversely, broader and less clearly-defined populations, such as “Community-dwelling elderly/N = 1,857,” ^10^ exhibited the lowest mean AUC value, of 0.79. This may reflect the inherent diversity of such groups, which often include individuals with varying health stages and comorbidities, thereby complicating the uniformly-effective application of pathology detection methods.

The results analyzed in the current review indicate that blood-based p-tau217 is one of the most promising biomarkers currently available for the diagnosis and screening of AD. Its diagnostic accuracy, with AUC values consistently exceeding 0.90, is comparable to gold-standard methods such as CSF analysis and PET-tau imaging. 12 13 15 This high performance is observed in multiple scenarios, including patients with initial cognitive complaints, carriers of genetic mutations, and asymptomatic individuals. 6 12 16

One of the most significant advances is the validation of p-tau217 in ethnically-diverse populations and in primary-care settings. The study by Sanford et al. 8 demonstrated that p-tau217 maintains its high diagnostic accuracy in White, Black, and Latino individuals, outperforming other biomarkers and proving to be a more equitable tool. Additionally, its validation in primary-care cohorts, as conducted by Ashton et al. 17 , confirms its potential to transform the initial screening for dementia, enabling general practitioners to safely identify patients who require specialized evaluation.

## DISCUSSION

Beyond early detection, the ability of p-tau217 to differentiate AD from other dementias, such as FTD, is a standout feature. 14 This is crucial for clinical management and eligibility for specific therapies. A new horizon for p-tau217 is its role in monitoring therapeutic interventions. Recent studies 18 with anti-amyloid therapies (such as lecanemab) have shown that plasma p-tau217 levels decrease in response to treatment, correlating with the removal of amyloid plaques. This positions the biomarker as a diagnostic tool and as a potential marker of therapeutic response.

Despite the robust evidence, the transition to routine clinical practice requires overcoming methodological and logistical challenges. 19 Standardization of assays and cutoff values across different laboratory platforms is essential to ensure the comparability of results. 4 Preanalytical factors, such as the time between sample collection and processing, can also influence results and require rigorous protocols. 20 Furthermore, longitudinal multicenter validation remains crucial to understand the biomarker's temporal curve and its interaction with comorbidities that may affect protein metabolism. 21

The higher diagnostic accuracy observed in primary-care cohorts compared with longitudinal studies may reflect differences in disease spectrum and case enrichment. Primary-care settings often include symptomatic individuals with higher pretest probability, enhancing biomarker performance. In contrast, longitudinal population-based cohorts include heterogeneous individuals across disease stages, introducing variability and reducing apparent accuracy. Additionally, longer follow-up periods may introduce biological and methodological variability, influencing predictive performance.

Despite evidence of consistent performance across White, Black, and Latino populations, there remains a critical gap in representation from low- and middle-income countries (LMICs), as well as regions such as Africa and parts of Asia. This limitation restricts the generalizability of findings and highlights the need for global validation studies in diverse healthcare and socioeconomic contexts.

The current review has limitations. First, the integrative design does not enable a quantitative synthesis such as pooled sensitivity and specificity. Second, heterogeneity in study design, assay methods, and populations limits direct comparability. Third, potential publication bias cannot be excluded. Finally, despite updating the search, the rapidly-evolving nature of this field means that new evidence may emerge shortly after publication.

The findings of the present review underscore that, while plasma p-tau217 is a revolutionary diagnostic tool, its transition from a research environment to a bedside reality requires addressing several physiological and technical complexities. Biological confounders and clinical limitations: Despite the high diagnostic accuracy observed across diverse cohorts, several patient-specific factors can influence plasma p-tau217 levels, potentially leading to clinical misinterpretation. Chronic kidney disease (CKD): Renal function plays a critical role in the clearance of tau fragments from the bloodstream. Recent evidence 22 23 24 25 indicates that individuals with an estimated glomerular filtration rate (eGFR) (CKD stage 3b or higher) exhibit significantly elevated plasma p-tau217 levels, which may lead to inaccurate classification of AD pathology if not adjusted for renal health. Diurnal variation: Emerging research 23 has identified a significant time-of-day effect on biomarker concentrations. For p-tau217, the lowest levels are typically observed in the morning, upon waking, with the highest values occurring in the afternoon or early evening. This variation is of a magnitude similar to that of the annual increase in at-risk individuals, suggesting that standardized collection times are essential for longitudinal monitoring. Hemoglobin and anemia: Systemic factors such as hemoglobin levels can impact biomarker readings. Clinical studies 23 have shown that anemia is cross-sectionally associated with higher levels of plasma p-tau217, potentially reflecting an interplay between chronic cerebral hypoxia and neurodegenerative pathology. Body Mass Index (BMI): While p-tau217 is more robust than other biomarkers to metabolic changes, high BMI remains a potential confounder due to blood-volume expansion and the “dilution effect,” which may subtly lower absolute concentrations in obese individuals compared with those with lower BMI. Assay heterogeneity and platform standardization: A significant barrier to universal implementation is the inherent heterogeneity among available testing platforms. Currently, the evidence base relies on several primary analytical platforms, including Lilly, MSDQuanterix, Simoa, ALZpath, and Olink. 24 It is critical to recognize that these platforms use different antibodies and signal-detection technologies, producing non-interchangeable absolute concentrations. For instance, a cut-off established on the ALZpath assay cannot be directly applied to results from a Simoa-based assay. This necessitates the development of platform-specific cut-offs and international reference standards to ensure that a diagnosis is consistent regardless of the laboratory equipment used. 22 23 24 25

In summary, the joint and detailed assessment of the data and graphs highlights the intrinsic interdependence regarding study design, the characteristics of the investigated population, and the achieved accuracy (AUC) in pathology detection. The numerical AUC values provide quantitative evidence supporting the conclusion that optimizing diagnostic accuracy requires a strategic harmonization regarding the choice of appropriate research designs, taking into account the specificities of the pathology under investigation, and the selection of relevant and well-defined population groups whose intrinsic characteristics may influence the sensitivity and specificity of detection methods.

In conclusion, plasma p-tau217 represents a breakthrough in the search for non-invasive, accessible, and reliable biomarkers for AD. Its diagnostic performance is comparable to that of gold-standard tests, with the advantage of being applicable in diverse populations and primary-care contexts. Its usefulness extends to screening asymptomatic individuals, etiologically differentiating among dementias, and, promisingly, monitoring therapeutic response.

The incorporation of p-tau217 into the clinical practice could transform early diagnosis, enabling interventions at earlier stages and promoting greater accessibility and equity. However, to consolidate its use, technical standardization, continuous validation, and overcoming implementation challenges are essential steps. Therefore, p-tau217 emerges not just as a promising marker, but as a potential game-changer in the diagnosis and early management of AD.

**Figure 2:**
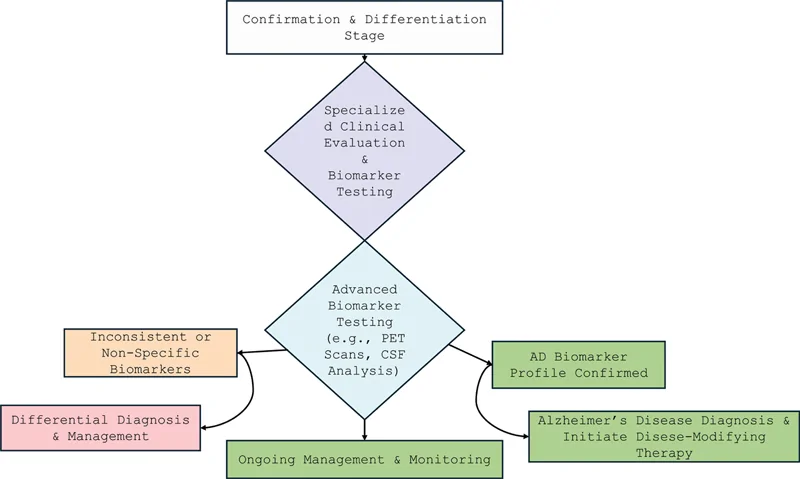
Diagnostic and clinical pathway using plasma p-tau217 for Alzheimer's disease.

## References

[1] Morris J C Roe C M Xiong C Plasma phospho-tau217 and phospho-tau181 as biomarkers in Alzheimer's disease: a prospective, longitudinal cohort study Lancet Neurol 2023 22 01 66 77 10.1016/S1474-4422(22)00409-7

[2] Mattsson-Carlgren N Salvadó G Ashton N J Prediction of Longitudinal Cognitive Decline in Preclinical Alzheimer Disease Using Plasma Biomarkers JAMA Neurol 2023 80 04 360 369 10.1001/jamaneurol.2022.5272 PMC10087054 36745413

[3] Zetterberg H Moving fluid biomarkers for Alzheimer's disease from research tools to routine clinical diagnostics Mol Neurodegener 2023 18 01 9 10.1186/s13024-023-00603-1 PMC7893769 33608044

[4] Li D Mielke M M An update on blood-based markers of Alzheimer's disease using the Simoa platform Neurol Ther 2019 8(Suppl 2):73 82 10.1007/s40120-019-00164-5 PMC6908531 31833025

[5] Palmqvist S Janelidze S Quiroz Y T Discriminative Accuracy of Plasma Phospho-tau217 for Alzheimer Disease vs Other Neurodegenerative Disorders JAMA 2020 324 08 772 781 10.1001/jama.2020.12134 PMC7388060 32722745

[6] Ashton N J Brum W S Di Molfetta G Diagnostic Accuracy of a Plasma Phosphorylated Tau 217 Immunoassay for Alzheimer Disease Pathology JAMA Neurol 2024 81 03 255 263 10.1001/jamaneurol.2023.5319 PMC10804282 38252443

[7] Li Y Schelke M W Sundström A Diagnostic and prognostic utility of plasma p-tau217 and p-tau181 in Alzheimer's disease and related dementias Alzheimers Res Ther 2024 16 01 50 10.1186/s13195-024-01588-2

[8] Mielke M M Dage J L Frank R D Performance of plasma phosphorylated tau 181 and 217 in the community Nat Med 2022 28 07 1398 1405 10.1038/s41591-022-01822-2 PMC9329262 35618838

[9] Thijssen E H La Joie R Strom A Plasma phosphorylated tau 217 and phosphorylated tau 181 as biomarkers in Alzheimer's disease and frontotemporal lobar degeneration: a retrospective diagnostic performance study Lancet Neurol 2021 20 09 739 752 10.1016/S1474-4422(21)00214-3 PMC8711249 34418401

[10] Xiao Z Zhou X Zhao Q Cao Y Ding D Significance of plasma p-tau217 in predicting long-term dementia risk in older community residents: Insights from machine learning approaches Alzheimers Dement 2024 20 10 7037 7047 10.1002/alz.14178 PMC11485078 39115912

[11] Khalafi M Dartora W J McIntire L BJ Diagnostic accuracy of phosphorylated tau217 in detecting Alzheimer's disease pathology among cognitively impaired and unimpaired: A systematic review and meta-analysis Alzheimers Dement 2025 21 02 e14458 10.1002/alz.14458 PMC11848338 39711334

[12] Janelidze S Berron D Smith R Associations of plasma phospho-tau217 levels with tau positron emission tomography in early Alzheimer disease JAMA Neurol 2021 78 02 149 156 10.1001/jamaneurol.2020.4201 PMC7653537 33165506

[13] Therriault J Servaes S Tissot C Equivalence of plasma p-tau217 with cerebrospinal fluid in the diagnosis of Alzheimer's disease Alzheimers Dement 2023 19 11 4967 4977 10.1002/alz.13026 PMC10587362 37078495

[14] Benussi A Huber H Tan K Plasma p-tau _217_ and neurofilament/p-tau _217_ ratio in differentiating Alzheimer's disease from syndromes associated with frontotemporal lobar degeneration Alzheimers Dement 2025 21 02 e14482 10.1002/alz.14482 PMC11848195 39776166

[15] Zhong X Wang Q Yang M Plasma p-tau217 and p-tau217/Aβ1-42 are effective biomarkers for identifying CSF- and PET imaging-diagnosed Alzheimer's disease: Insights for research and clinical practice Alzheimers Dement 2025 21 02 e14536 10.1002/alz.14536 PMC11848202 39887504

[16] Aguillon D Langella S Chen Y Plasma p-tau217 predicts in vivo brain pathology and cognition in autosomal dominant Alzheimer's disease Alzheimers Dement 2023 19 06 2585 2594 10.1002/alz.12906 PMC10271963 36571821

[17] Janelidze S Berron D Smith R Associations of Plasma Phospho-Tau217 Levels With Tau Positron Emission Tomography in Early Alzheimer Disease JAMA Neurol 2021 78 02 149 156 10.1001/jamaneurol.2020.4201 PMC7653537 33165506

[18] Mattsson-Carlgren N Janelidze S Palmqvist S Longitudinal plasma p-tau217 is increased in early stages of Alzheimer's disease Brain 2020 143 11 3234 3241 10.1093/brain/awaa286 PMC7719022 33068398

[19] Janelidze S Barthélemy N R Salvadó G Plasma Phosphorylated Tau 217 and Aβ42/40 to Predict Early Brain Aβ Accumulation in People Without Cognitive Impairment JAMA Neurol 2024 81 09 947 957 10.1001/jamaneurol.2024.2619 PMC11284634 39068669

[20] Hansson O Edelmayer C E Boxer A L The Alzheimer's Association appropriate use recommendations for blood biomarkers in Alzheimer's disease Alzheimers Dement 2022 18 12 2669 2686 10.1002/alz.12756 PMC10087669 35908251

[21] Teunissen C E Verberk I MW Thijssen E H Blood-based biomarkers for Alzheimer's disease: towards clinical implementation Lancet Neurol 2022 21 01 66 77 10.1016/S1474-4422(21)00361-6 34838239

[22] Palmqvist S Whitson H E Allen L A Alzheimer's Association Clinical Practice Guideline on the use of blood-based biomarkers in the diagnostic workup of suspected Alzheimer's disease within specialized care settings Alzheimers Dement 2025 21 07 e70535 10.1002/alz.70535 PMC12306682 40729527

[23] Mohaupt P Kindermans J Vialaret J Blood-based biomarkers and plasma Aβ assays in the differential diagnosis of Alzheimer's disease and behavioral-variant frontotemporal dementia Alzheimers Res Ther 2024 16 01 279 10.1186/s13195-024-01647-w PMC11687143 39736793

[24] Valletta M Vetrano D L Qiu C Anemia and Blood Biomarkers of Alzheimer Disease in Dementia Development JAMA Netw Open 2026 9 04 e264029 10.1001/jamanetworkopen.2026.4029 PMC13090852 41996117

[25] Bornhorst J A Lundgreen C S Weigand S D Quantitative Assessment of the Effect of Chronic Kidney Disease on Plasma P-Tau217 Concentrations Neurology 2025 104 03 e210287 10.1212/WNL.0000000000210287 PMC11744813 39823562

